# Synthesis of New 4-Aminoquinolines and Evaluation of Their *In Vitro* Activity against Chloroquine-Sensitive and Chloroquine-Resistant *Plasmodium falciparum*

**DOI:** 10.1371/journal.pone.0140878

**Published:** 2015-10-16

**Authors:** Chandima S. K. Rajapakse, Maryna Lisai, Christiane Deregnaucourt, Véronique Sinou, Christine Latour, Dipankar Roy, Joseph Schrével, Roberto A. Sánchez-Delgado

**Affiliations:** 1 Chemistry Department of Brooklyn College and Ph.D. Program in Chemistry, The Graduate Center of The City University of New York, New York, New York, United States of America; 2 Unité Molécules de Communication et Adaptation des Microorganismes, (MCAM, UMR 7245), Sorbonne Universités, Muséum National d’Histoire Naturelle CNRS, Paris, France; 3 Laboratory of Parasitology, Faculty of Pharmacy, UMR-MD3, Aix-Marseille University, Marseille, France; RWTH Aachen University, GERMANY

## Abstract

The efficacy of chloroquine, once the drug of choice in the fight against *Plasmodium falciparum*, is now severely limited due to widespread resistance. Amodiaquine is one of the most potent antimalarial 4-aminoquinolines known and remains effective against chloroquine-resistant parasites, but toxicity issues linked to a quinone-imine metabolite limit its clinical use. In search of new compounds able to retain the antimalarial activity of amodiaquine while circumventing quinone-imine metabolite toxicity, we have synthesized five 4-aminoquinolines that feature rings lacking hydroxyl groups in the side chain of the molecules and are thus incapable of generating toxic quinone-imines. The new compounds displayed high *in vitro* potency (low nanomolar IC_50_), markedly superior to chloroquine and comparable to amodiaquine, against chloroquine-sensitive and chloroquine-resistant strains of *P*. *falciparum*, accompanied by low toxicity to L6 rat fibroblasts and MRC5 human lung cells, and metabolic stability comparable or higher than that of amodiaquine. Computational studies indicate a unique mode of binding of compound **4** to heme through the HOMO located on a biphenyl moeity, which may partly explain the high antiplasmodial activity observed for this compound.

## Introduction

**Fig 1:**
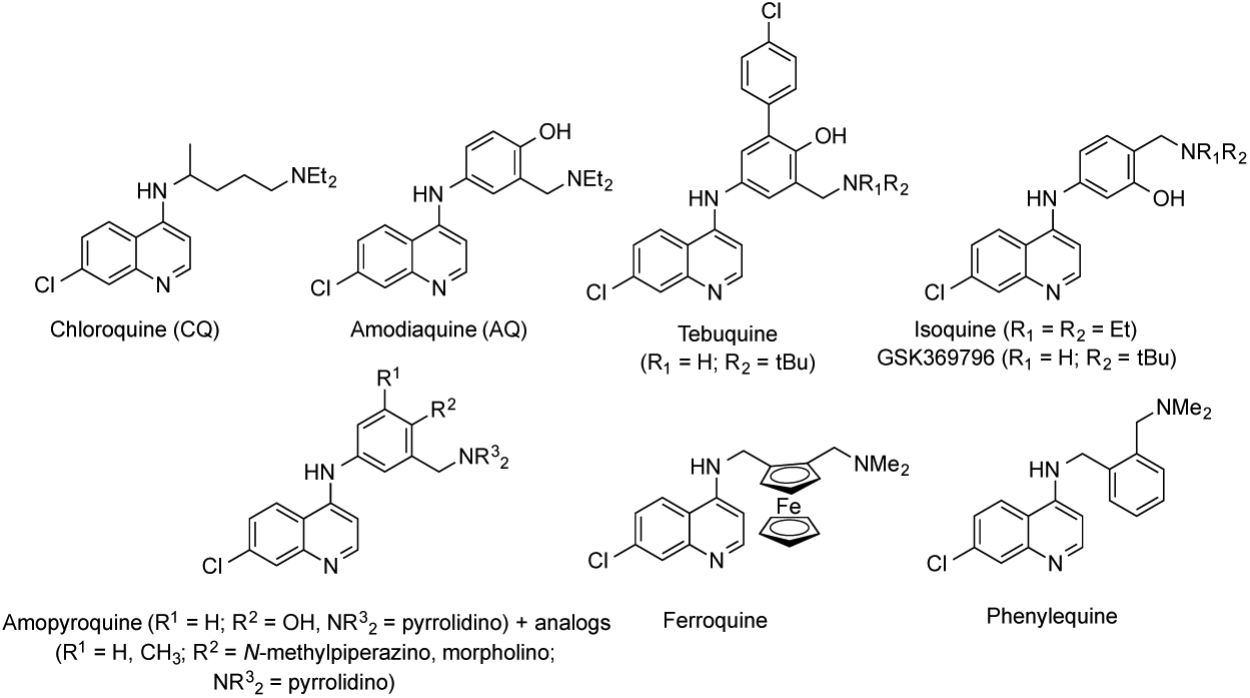
Aminoquinoline antimalarial drugs and drug candidates.

**Fig 2:**
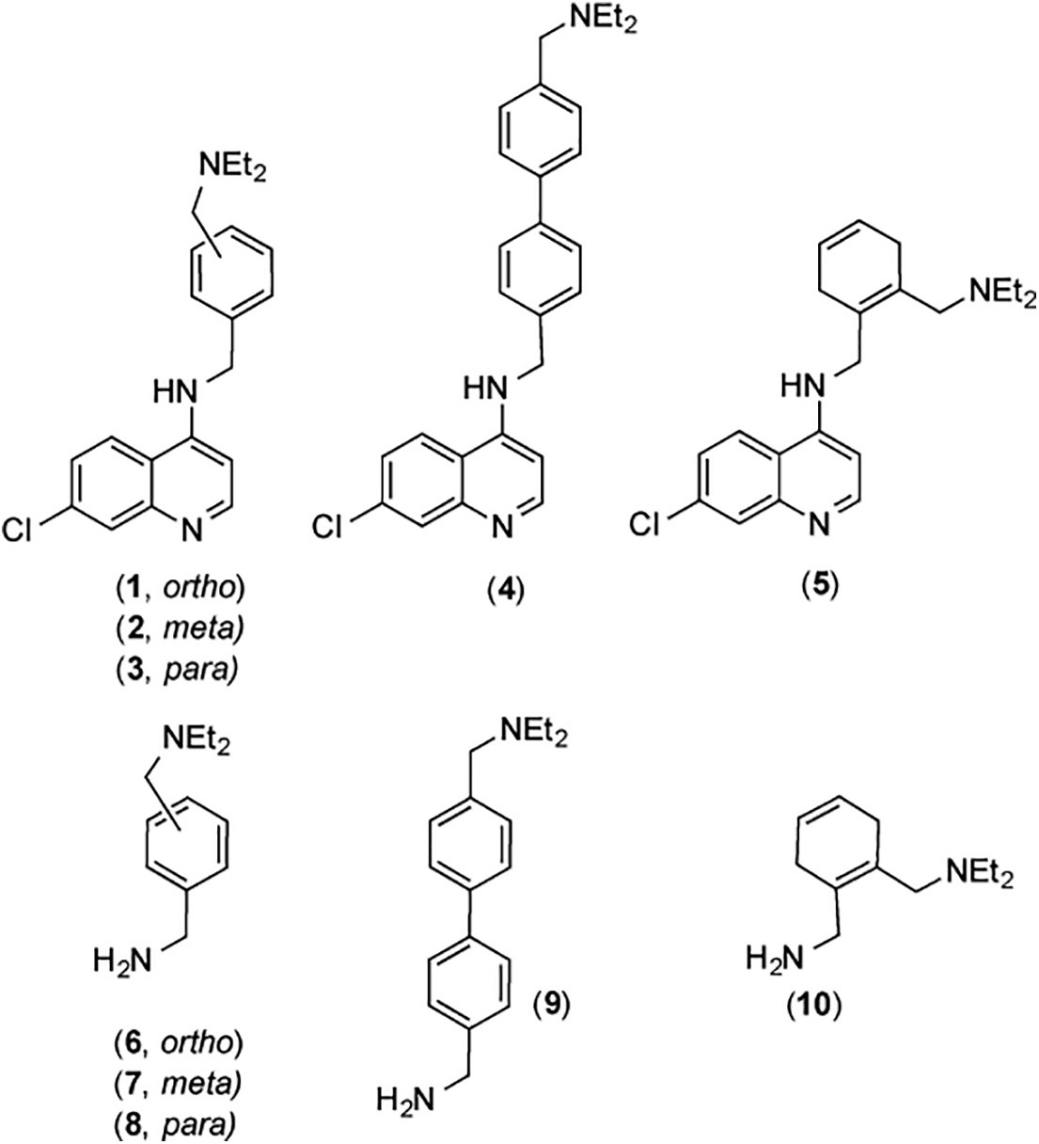
New aminoquinolines (1–5) and precursor amines (6–10) synthesized.

Malaria continues to be a major global health problem. According to the WHO, an estimated 3.2 billion people are at risk of being infected with *Plasmodium* and developing disease, and 1.2 billion are at high risk. It is estimated that 198 million cases of malaria occurred globally in 2013, leading to 584,000 deaths, mainly in the African Region (90% of all malaria deaths), and mostly in children under 5 years of age (78% of deaths) [1]. For decades one of the most successful and widely used drugs for treating malaria, especially *Plasmodium falciparum* infection, was chloroquine (CQ, Fig 1); however, widespread resistance has rendered it essentially useless in most parts of the world [2]. The 4-aminoquinoline pharmacophore has been the focus of intense efforts to develop new drugs not susceptible to resistance; for instance, amodiaquine (AQ, Fig 1), which is structurally related to CQ but contains a *p*-hydroxyanilino ring in the side chain of the molecule, is considerably more potent than CQ and remains effective against most CQ-resistant strains. However, toxicity issues limit its clinical use, in particular the occurrence during prolonged treatment or prophylaxis of agranulocytosis and potentially fatal idiosyncratic hepatotoxicity, linked to the formation of a reactive quinone-imine metabolite [3–7]. Related compounds like tebuquine (Fig 1) and its analogs also display high activity against CQ-resistant *P*. *falciparum* but are equally susceptible to P450-induced oxidation to toxic quinone-imine metabolites [8]. In order to circumvent this particular type of toxicity, other AQ analogs unlikely to form quinone-imine intermediates have been considered. They include isoquine [5,9] and the related GSK369796 [6,9]; amopyroquines [10–12] (Fig 1) and fluoroamodiaquines [13]; *N*-*tert*-butylamino Mannich base derivatives [14,15], and benzoxazines [4], all of which display high antiplasmodial activity. Other important developments concerning quinoline antimalarials are the discovery of ferroquine, a highly active and selective organometallic agent against CQ-resistant *P*. *falciparum* [16], and of phenylequine, a compound closely related to the ones described in this paper, which displays activity comparable to that of ferroquine against CQ-resistant parasites (Fig 1) [17]. Other aminoquinolines [18–24] and organometallic CQ derivatives [25–27] with interesting antimalarial properties have been reported in recent times. In this paper we describe the synthesis and antiplasmodial evaluation of a group of new 4-aminoquinolines 1–5 (Fig 2) structurally related to AQ, but lacking the 4-hydroxyl group in the side ring. These compounds are highly active *in vitro* against CQ-sensitive and CQ-resistant *P*. *falciparum* and are incapable of generating toxic quinone-imine metabolites.

## Results and Discussion

### Synthesis and characterization of new aminoquinolines

**Fig 3:**
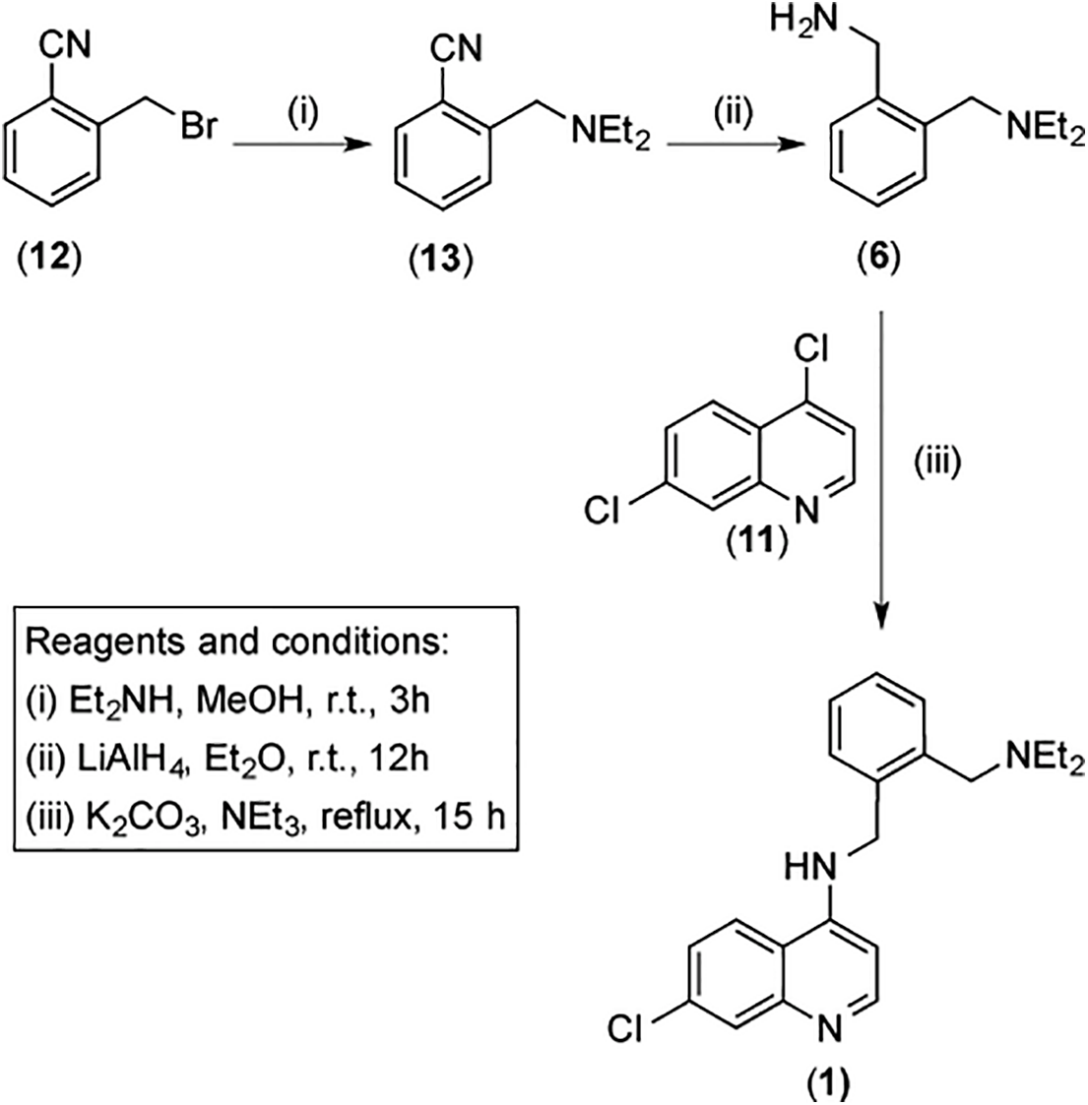
Synthetic strategy for compound 1 and other new aminoquinolines.

The new *N*-benzyl-4-aminoquinolines **1**–**4**, and the reduced *N*-cyclohexadienylmethyl derivative **5** (Fig 2) were prepared by condensation of the appropriate amines **6**–**10** (Fig 2) with 4,7-dichloroquinoline (**11**) in N-methyl-2-pyrrolidone (NMP) in the presence of K_2_CO_3_ and triethylamine, as exemplified in Fig 3 for compound **1**. The precursor amine **6** was obtained from the reaction of *o*-cyanobenzylbromide (**12**) with diethylamine in ethanol to yield *o*-(diethylaminomethyl)benzonitrile (**13**), followed by LiAlH_4_ reduction of the nitrile group in diethylether. The other (diethylaminomethyl)benzylamines used in this study (**7**–**9**) were prepared by analogous procedures, starting from the corresponding cyanobenzylbromides. Cyclohexadiene **10** was obtained by Birch reduction of **6**. All new compounds were characterized by ^1^H and ^13^C NMR spectroscopy and high-resolution mass spectrometry (complete data in Materials and Methods Section); the purity of all samples used in biological tests (> 95%) was established by elemental analysis and HPLC. It is worth noting that our high yield synthetic method for **1**–**5** relies on inexpensive commercially available starting materials.

### *In vitro* evaluation of antiplasmodial activity and cytotoxicity

The activity of compounds **1**–**5**, as well as CQ and AQ, was evaluated *in vitro* against the CQ-sensitive 3D7 and CQ-resistant K1 and Dd2 strains of *P*. *falciparum* in two independent laboratories designated as A and B. The results of these assays are collected in Table 1, where we note that the IC_50_ values for CQ toward the Dd2 and K1 from laboratory B were consistently lower than those from laboratory A. There are precedents for this type of differences in antiplasmodial activity as a function of the experimental conditions; more specifically, results of chemosusceptibility tests have been shown to be affected by the initial parasitemia, hematocrit, incubation time, time when ^3^H-hypoxanthine is added, the use of serum substitute, and the gas mixture [28]. Oxygen tension is a particularly important factor governing CQ activity against *P*. *falciparum*; it has been shown that high O_2_ tension (21%) leads to increased efficacy of the drug compared to lower (10%) O_2_ tension. Furthermore, the O_2_ influence was found to be strain-specific, with particular ocurrence in resistant strains [29]. In accordance with this, for Dd2 and K1 IC_50_ values for CQ from laboratory A, using an incubator with a fixed atmosphere of 5% CO_2_, 10% O_2_, 85% N_2_ for parasite culture, are higher than those from laboratory B, which employs a candle jar with a ~17% O_2_, 3% CO_2_, and 80% N_2_, whereas IC_50_ values for 3D7 are similar in both settings.

**Table 1:** *In* vitro activities of compounds 1–5, CQ, and AQ against CQ-sensitive and CQ-resistant strains of *P*. *falciparum*.

	Antiplasmodial activity, IC_50_ (nM)			Resistance Indexes	
	3D7	Dd2^b^	K1	Dd2/3D7	K1/3D7
			**A**		
CQ	17.6±0.71	521.6±95.73	1086.0±163.50	29.7	61.7
AQ	24.6±3.06	31.6±7.77	34.3±0.58	1.3	1.4
**1**	13.5±0.71	21.3±2.52	52.7±0.99	1.6	3.9
**2**	18.4±0.66	26.5±5.07	64.2±6.16	1.4	3.5
**3**	17.3±0.58	30.5±2.12	60.3±4.07	1.8	3.5
**4**	15.2±1.57	20.7±0.20	53.4±2.03	1.4	3.5
**5**	-	59.0±1.85	63.7±1.46	-	-
			**B**		
CQ	18.0±12.31	35.1±12.08	124.0±48.05	1.9	6.9
AQ	11.9±1.00	11.3±0.64	9.60±0.51	0.9	0.8
**1**	21.6±3.11	19.4±2.18	14.6±0.36	0.9	0.7
**2**	17.1±2.97	17.1±1.72	12.0±1.15	1.0	0.7
**3**	16.1±2.44	17.7±3.78	12.4±0.40	1.1	0.8
**4**	12.9±0.85	14.4±0.50	11.8±1.00	1.1	0.9
**5**	-	-	20.2±6.55	-	-

IC_50_ ± SD values (nM) determined from independent experiments performed in triplicate in two laboratories (**A**, Marseille; **B**, Paris) under different assay conditions (see main text and Materials and Methods Section for details). CQ: chloroquine, AQ: amodiaquine, -: not determined.

^b^Note that Dd2 is CQ-resistant under the culture conditions of laboratory A and CQ-sensitive under the culture conditions of laboratory B.

The new compounds **1**–**5** display high activity against all the parasites assayed. For the CQ-sensitive strain 3D7, **1**–**4** show comparable or slightly higher potency than those measured for CQ and AQ. More importantly, when tested against the CQ-resistant parasites (K1 and Dd2) the activity of **1**–**5** is consistently much higher than that of CQ. Compound **1**, which only differs from the known highly active phenylequine [17] in that it contains a diethylamino instead of dimethylamino group at the end of the side chain, was about 21 times more potent than CQ against K1 parasites under conditions A, and close to eigth times more active under conditions B. Switching to the *meta* and *para* isomers **2** and **3**, or replacing the arene ring by the corresponding cyclohexadiene in **5** led to a slight decrease of activity under conditions A and a slight increase under conditions B. On the other hand, the presence of the large biphenyl group in **4** resulted in activity as high as that of **1** under conditions A, and in the highest potency of all compounds against K1 under conditions B. Compounds **1**–**5** are also more effective than CQ against the Dd2 strain, which was found to be CQ-resistant under conditions A but CQ-sensitive under conditions B. The most active compounds are **1** and **4** also in this case, with the latter again being the most potent under both sets of conditions A and B. Additional antiplasmodial activity data for selected compounds against CQ-sensitive (F32) and CQ-resistant (K14 and FcB1) strains are collected in S1 Table (Supporting Information). The trends observed in those cases are similar to the ones described above, with the new compounds being comparable or somewhat better than CQ against CQ-sensitive parasites and much more active than CQ against resistant strains. Compound **4** showed once more the highest potency against the highly resistant K14 strain under conditions A, with a remarkable IC_50_ of 7.5 nM.

The low values of the resistance indexes (RI = IC_50_Dd2/IC_50_3D7 and IC_50_K1/IC_50_3D7) for **1**–**4** suggest a low potential for these compounds to develop cross-resistance with CQ [18,23]. It is also important to highlight from the data in Table 1 and S1 Table that the antimalarial potency of the new 4-aminoquinolines against CQ-resistant strains of *P*. *falciparum* is similar to that of the highly active AQ. As noted above, the clinical use of AQ has been hampered by its tendency to form toxic quinone-imine metabolites. The molecular design of the compounds presented here eliminates the potential for this type of toxicity, since all of them lack the 4-hydroxyl group in the ring present in the side chain of each molecular structure and are therefore incapable of generating quinone-imine intermediates.

In order to further ascertain the possibility of indiscriminate cytotoxicity we also measured the ability of compounds **1**–**4** to inhibit normal rat L6 and human MRC5 cell lines by the method described in the Materials and Methods Section. The data in Table 2 reveal low toxicity to the mammalian cells, which translates into high selectivity ranges for both CQ-sensitive and CQ-resistant *P*. *falciparum*.

**Table 2:** Cytotoxicity of compounds 1–4 toward mammalian cells.

	L6			MRC5		
	CC_50_ ^a^(μM)	SI^b^(min-max)		CC_50_(μM)	SI(min-max)	
		CQS strains	CQR strains		CQS strains	CQR strains
**1**	27.7 ± 1.73	923–2052	442–2289	26.4 ± 1.75	880–1955	421–2182
**2**	20.6 ± 3.92	1505–1884	431–1544	20.3 ± 3.40	1435–1796	411–1544
**3**	13.8 ± 1.90	1140–1720	459–1565	13.0 ± 2.26	1086–1639	438–1491
**4**	2.8 ± 0.15	1639–2147	519–3693	2.6 ± 0.12	1562–2046	494–3520

^*a*^CC_50_: concentration of drug inducing 50% of cell growth arrest.

^*b*^SI: Selectivity index expressed as (CC_50_ to each mammalian cell line)/(IC_50_ to *P*. *falciparum*). SI max corresponds to the most sensitive strain of *P*. *falciparum* and SI min toward the less sensitive parasite strain.

### *In vitro* metabolic stability measurements

We also examined the metabolic stability of compounds **1**–**4**, as well as of CQ and AQ, by measuring their intrinsic clearance and half-lives upon incubation with human liver microsomes in the presence and in the absence of NADPH. The data (Table 3) were compared with values for the well-characterized positive controls verapamil (CL_int_ 134 mL min^-1^ mg^-1^, t_1/2_ 17.2 min) and warfarin (CL_int_ 0.0 mL min^-1^ mg^-1^, t_1/2_ >240 min), which are considered of low and high metabolic stability, respectively.

**Table 3:** Metabolic stability data.

Compd	NADPH-dep. ^a^CL_int_ (μl.min^-1^.mg^-1^)	NADPH-dep. ^b^t_1/2_ (min)	NADPH-free ^b^t_1/2_ (min)
**1**	45.6±0.12	50.9±0.55	>240
**2**	56.2±0.55	41.1±0.41	>240
**3**	51.1±3.06	45.3±1.36	>240
**4**	465.9±43.99	5.0±0.47	>240
CQ	17.5±2.03	133.2±15.52	>240
AQ	431±33.4	5.4±0.42	>240

[Compd] = 1 μM. [protein] = 0.3 mg/mL.

^a^Microsomal Intrinsic Clearance.

^b^Half-life

In the absence of NADPH no significant metabolism was observed for any of the compounds tested (t_1/2_ > 240). In the presence of NADPH, CQ displayed the longest half-life of all the aminoquinolines (133 ± 15.5 min), while AQ showed a short half-life of 5.4 ± 0.42 min. The t_1/2_ values for **1**–**3** are in the range 40–51 min, intermediate between CQ and AQ, while compound **4**, which appears as the most active of the series, shows a t_1/2_ ~5 min, very similar to that of AQ. It is known that CQ [30] is rapidly dealkylated via cytochrome P450 enzymes into the pharmacologically active desethylchloroquine (DECQ), and to a lesser extent, bisdesethylchloroquine, (BDECQ), while AQ is quickly metabolized into active desethylamodiaquine (DEAQ); elimination of the active metabolites is in turn very slow (in the particular case of the rapidly metabolized AQ the terminal half-life is ~100 h) [31]. Therefore both CQ and AQ may be considered pro-drugs, which are bio-activated to DECQ and DEAQ; it is likely that **1**–**4** are metabolized by P450 enzymes in a similar manner.

### Computational studies

**Fig 4:**
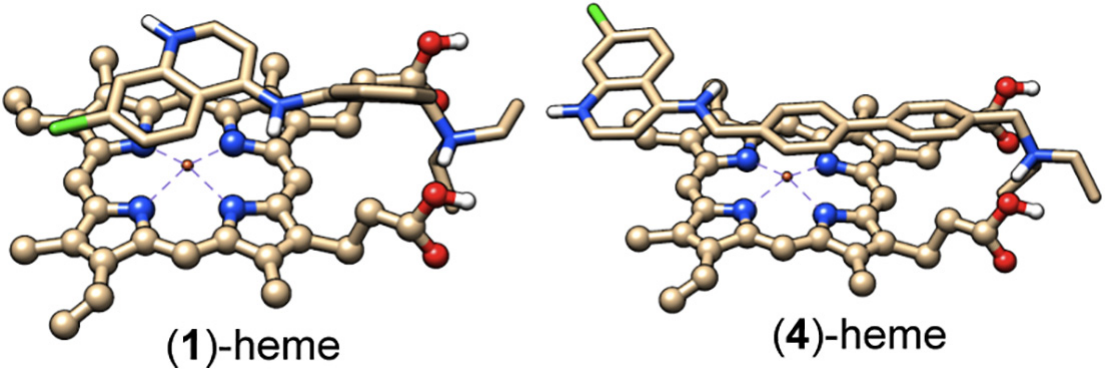
Docked poses of 1 and 4 on heme. Both aminoquinoline molecules were employed in the diprotonated form. Atom color code: white: H, brown: C, blue: N, red: O, and gold: Fe. Only polar hydrogens are shown for clarity.

**Fig 5:**
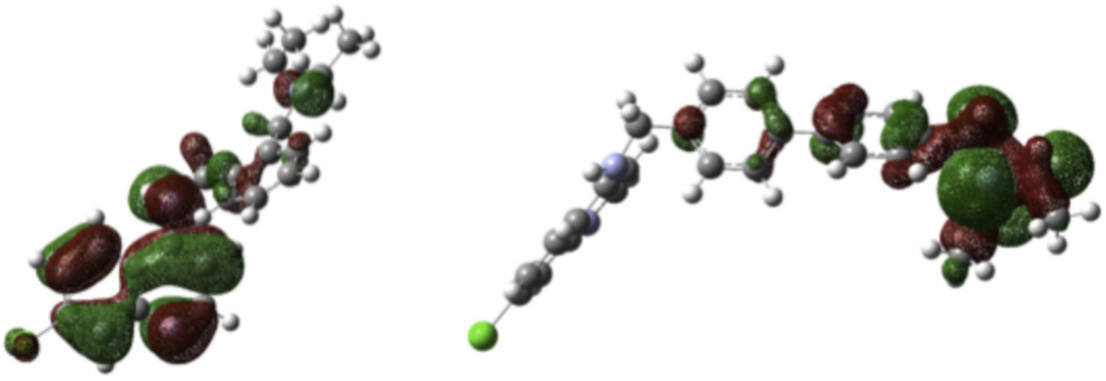
HOMO’s of compounds 1 and 4.

Aminoquinoline drugs exert their antimalarial action by disrupting aggregation of heme into hemozoin. It is therefore interesting to examine the interactions of new drug candidates with heme, to try to find correlations with antimalarial potency. The binding of **1**–**5**, CQ, and AQ to heme was analyzed by use of Autodock 4.2.11 (Details in Materials and Methods Section). For all the compounds studied the energetically most favorable binding is guided by H-bonding as well as π-stacking interactions between the aminoquinoline and heme. Although the docking energies shown in Table 4 are comparable for compounds **1**–**5** and only marginally stronger than for CQ, a significant difference in the nature of the interactions among the molecules studied is that **s**tacking of **4** over the porphyrin takes place through the biphenyl moiety, whereas for all other systems the stacking happens via the quinoline ring (Fig 4 and S1 Fig in Supporting Information). This different binding mode of **4** to heme is likely related to the localization of the HOMO over the biphenyl unit, unlike the other four molecules for which the HOMO is concentrated mostly over the quinoline moeity (Fig 5, and S2 Fig, Supporting Information). This structural difference could be in part responsible for the high activity observed for this compound.

**Table 4:** Computed docking energies.^a^

Compd	1	2	3	4	5	CQ	AQ
ΔE Docking (kcal/mol)^b^	-5.58	-5.11	-5.42	-5.97	-5.31	-4.72	-5.38

^a^Computed at the B3LYP/6-31G(d) level using the solvation model of Truhlar and co-workers. Computational details are found in the Supporting Information.

^b^Bis-protonated ligand geometries used for all docking simulations.

## Conclusion

We have synthesized a series of new 4-aminoquinolines designed to display the high antimalarial potency of amodiaquine while avoiding associated toxicity issues caused by quinone-imine metabolites. This molecular design led to compounds with high *in vitro* activity, markedly superior to chloroquine and comparable to amodiaquine, against CQ-sensitive and particularly against CQ-resistant strains of *P*. *falciparum*, accompanied by low toxicity toward L6 rat fibroblasts and MRC5 human lung cells. Compounds **1**–**3** display moderate *in vitro* metabolic stability to human liver microsomes, lower than that of chloroquine but higher than amodiaquine, while **4** shows a shorter half-life, very similar to that of amodiaquine. The high activity of **4** is possibly linked with a unique mode of bindig to heme through the biphenyl unit.

## Materials and Methods

### Synthesis of compounds

#### General

Solvents (analytical grade, Aldrich) were purified immediately prior to use by means of an Innovative Technology solvent purification unit. Other reagents (Aldrich) were used as received. NMR spectra were obtained at 400 MHz for ^1^H and 75 MHz for ^13^C using an AVANCE Bruker 400 instrument; δ values are referred to residual proton or carbon signals in the deuterated solvents. Elemental analyses were performed by Atlantic Microlab, Norcross, Georgia. The Mass Spectrometry Service of Hunter College CUNY performed the mass spectral analyses.

#### Precursor nitriles 13–16

***o*-(Diethylaminomethyl)benzonitrile (13):** Dropwise addition of *o*-cyanobenzylbromide (**12**) (10.0 g, 50 mmol) in ethanol (30mL) to cold diethylamine (57 mL, 0.55 mol) was followed by 3 h of stirring at room temperature. The solution became orange and a white precipitate was observed. Aqueous Na_2_CO_3_ (20 mL, 0.1 M) was added until the solution became basic to pH paper. At this time the white precipitate dissolved. Then the solution was concentrated and extracted into an ether layer. The organic layer was washed with water 3 times, dried over Na_2_SO_4_, and evaporated under vacuum to yield the product as a reddish-orange oil. Yield: 8.47 g, 90%. ^1^H NMR (CDCl_3_) δ (ppm): 7.54 (m, 2H); 7.47 (td, 1H); 7.22 (td, 2H); 3.69 (s, 2H); 2.49 (q, 4H); 0.98 (t, 6H). ESI-MS (M+H^+^), 189.1313 (calc), 189.1388 (found).

***m*-(Diethylaminomethyl)benzonitrile** (**14**) was prepared by following an analogous procedure, using *m*-cyanobenzylbromide (6.0 g, 30 mmol) in ethanol (40 mL) and cold diethylamine (35 mL, 340 mmol) and stirred at room temperature for 3 h. The product was collected as an orange-colored oil. Yield 4.1 g, 72%. ^1^H NMR (CDCl_3_) δ (ppm): 7.7 (s, 1H); 7.61 (d, 1H); 7.56 (d, 1H), 7.45 (t, 1H), 3.26 (s, 2H), 2.55 (q, 4H), 1.05 (t, 6H).

***p*-(Diethylaminomethyl)benzonitrile** (**15**) was prepared by following an analogous procedure, using *p*-cyanobenzylbromide (7.2 g, 36 mmol) in THF (25 mL) and cold diethylamine (40 mL, 403 mmol), stirring for 1.5 h at room temperature. The product was collected as brown-orange oil. Yield 5.9 g, 86%. ^1^H NMR (CDCl_3_) δ (ppm): 7.57 (m, 2H); 7.47 (m, 2H); 3.59 (s, 2H), 2.52 (q, 4H), 1.02 (t, 6H).

**4’-((diethylamino)methyl)-[1,1’biphenyl]-4-carbonitrile** (**16**) was prepared by following an analogous procedure, using 4’-bromomethyl-[1,1’-biphenyl]-4-carbonitrile (10.0 g, 36.7 mmol) in THF (40 mL) and cold diethylamine (40 mL, 403 mmol), stirring for 1.5 h at room temperature. The product was collected as a colorless oil. Yield 9.4 g, 97%. ^1^H NMR (CDCl_3_) δ (ppm): 7.8 (d, 1H); 7.65 (m, 1H); 7.55 (m, 6H), 3.65 (s, 2H), 2.6 (q, 4H), 1.1 (t, 6H).

#### Precursor amines 6–10

***o*-(Diethylaminomethyl)benzylamine (6)** *o*-(Diethylaminomethyl)benzonitrile **(13)** (6.0 g, 32 mmol) in anhydrous diethylether (20 mL) was added dropwise over a period of 10 min to a suspension of LiAlH_4_ (2.4 g, 64 mmol) in anhydrous diethyl ether (50 mL, 0°C, N_2_ atmosphere). The mixture was allowed to come to room temperature, stirred for 12 h, cooled in ice, treated dropwise with 20% NaOH (17 mL) with cooling, and extracted three times with ether (50 mL each). The combined organic phases were dried over Na_2_SO_4_, filtered, and evaporated under vacuum. The product was collected as reddish-orange colored oil. Yield 6.8 g, 79%. ^1^H NMR (CDCl_3_) δ (ppm): 7.17 (m, 4H); 3.75 (s, 2H); 3.50 (s 2H); 2.43 (q, 4H): 1.928 (s, NH_2_); 0.962 (t, 6H). ESI-MS (M+H^+^) = 193.1626 (calc), 193.1715 (found).

***m*-(Diethylaminomethyl)benzylamine (7)** was prepared by an analogous procedure using *m*-(diethylaminomethyl)benzonitrile (**14**) (4.1 g, 22 mmol) in anhydrous diethylether (15 mL) and lithium aluminum hydride (1.7 g, 44 mmol) in anhydrous diethyl ether (40 mL). The product was collected as orange colored oil. Yield 3.4 g, 81%, ^1^H NMR (CDCl_3_) δ (ppm): 7.29 (m, 4H); 3.86 (s, 2H); 3.56 (s, 2H), 3.26 (s, 2H), 2.54 (q, 4H), 1.04 (t, 6H).

***p*-(Diethylaminomethyl)benzylamine (8)** was prepared by an analogous procedure using *p*-(diethylaminomethyl)benzonitrile (**15**) (5.9 g, 32 mmol) in anhydrous diethylether (15 mL) and lithium aluminum hydride (2.4 g, 63 mmol) in anhydrous ether (15 mL) The product was collected as a pale orange colored oil. Yield 4.1 g, 67%. ^1^H NMR (CDCl_3_) δ (ppm): 7.27 (m, 4H); 3.84 (s, 2H); 2.52 (q, 4H), 1.03 (t, 6H).

***N*-((4’-(Aminomethyl)-[1,1’-biphenyl]-4-yl)methyl)-*N*,*N*-diethylamine (9)** was prepared by an analogous procedure using 4’-((diethylamino)methyl)-[1,1’biphenyl]-4-carbonitrile (**16**) (9.4 g, 36 mmol) in anhydrous diethylether (25 mL) and lithium aluminum hydride (2.7 g, 71 mmol) in anhydrous ether (25 mL). The product was collected as slightly orange colored oil. Yield 7.1 g, 78%. ^1^H NMR (CDCl_3_) δ (ppm): 7.3 (m, 8H); 3.83 (s, 2H); 3.64 (s, 2H), 2.58 (q, 4H), 1.1 (t, 6H).

***N*-((2-Aminomethyl)cyclohexa-1,4-dien-1-yl)methyl)-*N*,*N*-diethylamine (10)** A solution of *o*-(diethylaminomethyl)benzylamine **(6)** (1.5 g, 7.8 mmol) in anhydrous ethanol (24 mL) was added to liquid ammonia (25 mL) at –78°C. To this mixture was added Li (0.5 g, 10 eq.) in small portions over two hours. After quenching the reaction with NH_4_Cl (1.6 g) in H_2_O (7 mL), the aqueous suspension was extracted with CH_2_Cl_2_, and the organic extracts were dried (Na_2_SO_4_) and evaporated under vacuum to give the product as a yellow colored oil. Yield 1.3 g, 89%. ^1^H NMR (400 MHz, CDCl_3_) δ 5.72 (m 2H), 3.30 (s, 2H), 3.00 (s, 2H), 2.43 (q, 4H); 2.75 (m, 4H), 2.02 (s, NH_2_), 1.01 (t, 6H). ESI-MS (M+H^+^) = 195.1783 (calc), 195.1854 (found).

#### New 4-aminoquinolines

**7-Chloro-*N*-(2-((diethylamino)methyl)benzyl)quinolin-4-amine (1)** *o*-(Diethylaminomethyl)-benzylamine (**6**) (0.8 g, 4.2 mmol), 4-7-dichloroquinoline (**11**) (5.0 g, 25 mmol), K_2_CO_3_ (1.0 g, 7.25 mmol), anhydrous triethylamine (5 mL, 36 mmol) and anhydrous NMP (7 mL) were placed in a 25 mL round bottomed flask and heated under reflux under nitrogen for 15 h. The mixture was allowed to cool to room temperature before diluting with ethyl acetate. The product was washed 10 times with brine, and then washed 6 times with a large amount of water to remove NMP. The organic layer was dried over Na_2_SO_4_, filtered, and the solvent was removed under reduced pressure. The product was purified by column chromatography (ethylacetate: hexane: three drops of Et_3_N) and collected as a yellow crystalline product. Yield 0.7 g, 50%. ^1^H NMR (MeOD), δ (ppm): 8.20 (d, *J* = 5.6 Hz, 1H), 7.93 (d, *J* = 9.0 Hz,1 H); 7.68 (d, *J* = 1.7 Hz, 1H), 7.29 (dd, *J* = 9.0 Hz, *J* ^*’*^ = 1.9 Hz, 1H), 7.28–7.34 (m, 4H), 6.44 (d, *J* = 5.6 Hz, 1H), 4.59 (s, 2H), 3.57 (s, 2H), 2.48 (q, *J* = 7.1 Hz, 4H), 0.93 (t, *J* = 7.1 Hz, 6H), ^13^C NMR (CDCl_3_), δ (ppm): 152.2, 150.4, 149.5, 137.5, 137.4, 134.7, 132.2, 130.7, 128.4, 127.9, 127.5, 124.6, 122.6, 118.1, 99.2, 56.4, 47.1, 46.5, 10.5. Anal. Calcd for C_21_H_24_N_3_Cl: C, 71.27; H, 6.84; N, 11.87. Found: C, 71.19; H, 6.85; N, 11.82. ESI-MS (M+H^+^) = 354.16588 (calc), 354.17501 (found).

**7-Chloro-*N*-(3-((diethylamino)methyl)benzyl)quinolin-4-amine (2)** was prepared by an analogous procedure using *m*-(diethylaminomethyl)benzylamine (**7**) (3.4 g, 18 mmol), K_2_CO_3_ (3.0 g, 21 mmol), 4-7-dichloroquinoline (**11**) (20.8 g, 105 mmol), triethylamine (22 mL, 159 mmol) and NMP (25 mL). The resulting product (white powder) was dried under vacuum. Yield 2.9 g, 47%, ^1^H NMR (CDCl_3_), δ (ppm): 8.54 (d, *J* = 4.8 Hz, 1H); 7.99 (s, 1H); 7.71 (d, *J* = 5.0 Hz, 1H), 7.29 (m, 5H), 6.48 (d, *J* = 4.8 Hz, 1H), 4.54 (s, 2H), 3.59 (s, 2H), 2.54 (q, *J* = 7.1 Hz, 4H), 1.04 (t, *J* = 7.1 Hz, 6H). ^13^C NMR (CDCl_3_), δ (ppm): 152.1, 149.5, 149.2, 141.1, 137.1, 134.9, 128.8, 128.5, 128.2, 128.1, 120.9, 117.2, 99.7, 57.4, 47.7, 46.8, 11.7. Anal. Calcd for C_21_H_24_N_3_Cl: C, 71.27; H, 6.84; N, 11.87. Found: C, 71.14; H, 6.95; N, 11.73. ESI-MS (M+H^+^) = 354.16588 (calc), 354.17610 (found M+H^+^).

**7-Chloro-*N*-(4-((diethylamino)methyl)benzyl)quinolin-4-amine** (**3**) was prepared by an analogous procedure using *p*-(diethylaminomethyl)benzylamine (**8**) (4.1 g, 21 mmol), K_2_CO_3_ (3.5 g, 25.2 mmol), 4-7-dichloroquinoline (**11**) (20.8 g, 105 mmol), triethylamine (26 mL, 189 mmol) and NMP (30 mL). The resulting product (slightly yellow powder) was dried under vacuum. Yield 4.3 g, 57%, ^1^H NMR (CDCl_3_), δ (ppm): 8.58 (d, *J* = 3.9 Hz, 1H); 8.02 (s, 1H); 7.72 (d, *J* = 4.1 Hz, 1H), 7.30 (m, 4H), 7.41 (d, *J* = 4.0 Hz, 1H), 6.51 (d, *J* = 3.9 Hz, 1H), 4.53 (s, 2H), 3.62 (s, 2H), 2.58 (q, *J* = 7.0 Hz, 4H), 1.10 (t, *J* = 7.0 Hz, 6H). ^13^C NMR (CDCl_3_), δ (ppm): 152.1, 149.5, 149.1, 135.5, 134.9, 128.9, 127.5, 125.5, 120.9, 117.2, 99.7, 57.4, 47.7, 46.8, 11.7. Anal. Calcd for C_21_H_24_N_3_Cl: C, 71.27; H, 6.84; N, 11.87. Found: C, 71.07; H, 6.92; N, 11.83. ESI-MS (M+H^+^) = 354.16588 (calc), 354.17259 (found M+H^+^).

**7-Chloro-*N*-((4’-((diethylamino)methyl)-[1,1’-biphenyl]-4-yl)methyl)quinolin-4-amine (4)** was prepared by an analogous procedure using *N*-((4’-(aminomethyl)-[1,1’-biphenyl]-4-yl)methyl)-*N*,*N*-diethylamine (**9**) (7.8 g, 27.8 mmol), K_2_CO_3_ (3.1 g, 22.1 mmol), 4-7-dichloroquinoline (**11**) (14.6 g, 174 mmol), triethylamine (26 mL, 189 mmol), and NMP (30 mL). The resulting product (light yellow powder) was dried under vacuum. Yield 6.8 g, 56%. ^1^H NMR (CDCl_3_) δ (ppm): 8.49 (d, *J* = 8.2 Hz, 1H); 7.95 (s, 1H); 7.3 (m, 10H), 6.32 (d, *J* = 8.2 Hz, 1H), 4.49 (s, 2H), 3.6 (s, 2H), 2.54 (q, *J* = 7.2 Hz, 4H), 1.09 (t, *J* = 7.2 Hz, 6H). ^13^C NMR (CDCl_3_), δ (ppm): 152.0, 149.1, 141.9, 134.6, 128.0, 125.3, 120.9, 117.1, 99.5, 57.1, 46.8, 45.8, 11.7. Anal. Calcd for C_27_H_28_N_3_Cl: C, 75.42; H, 6.56; N, 9.77. Found: C, 75.12; H, 6.76; N, 9.81. ESI-MS (M+H^+^) = 430.19718 (calc), 430.20347 (found M+H^+^).

**7-Chloro-*N*-((2-((diethylamino)methyl)cyclohexa-1,4-dien-1-yl)methyl)quinolin-4-amine (5)** was prepared by an analogous procedure using *N*-((2-(aminomethyl)cyclohexa-1,4-dien-1-yl)methyl)-*N*,*N*-diethylamine (**10**) (0.9 g, 4.9 mmol), 4,7-dichloroquinoline (**11**) (5.0 g, 25 mmol), K_2_CO_3_ (1.0 g, 7.25 mmol), anhydrous triethylamine (5.0 mL, 36 mmol) and anhydrous NMP (7 mL). The product was isolated as a white crystalline solid. Yield 50%. ^1^H NMR (CDCl_3_): δ 8.45 (d, *J* = 2.2 Hz, 1H), 7.87 (d, *J* = 2.2 Hz, 1H), 7.63 (d, *J* = 8.9 Hz, 1H), 7.25 (dd, *J* = 8.9 Hz, *J* ^*’*^ = 2.1 Hz, 1H), 6.52 (s, NH), 6.36 (d, *J* = 5.50 Hz, 1H), 5.65 (d, *J* = 1.24 Hz, 2H), 3.78 (d, *J* = 4.24 Hz, 2H), 2.98 (s, 2H), 2.80 (m, 4H), 2.50 (q, *J* = 7.1 Hz, 4H), 0.954 (t, *J* = 7.1 Hz, 6H). ^13^C NMR (CDCl_3_): δ 152.1, 150.5, 149.3, 134.7, 132.2, 129.8, 128.7, 124.8, 124.5, 123.9, 121.8, 117.7, 99.0, 55.1, 46.8, 45.5, 32.3, 31.7, 11.1. Anal. Calcd for C_21_H_26_N_3_Cl: C, 70.87; H, 7.36; N, 11.81.Found: C, 70.71; H, 7.39; N, 11.70. ESI-MS (M+H^+^) = 356.1815 (calc), 356.1877 (found).

### Biological Assays

#### Antimalarial Activity Measurements

The following strains of *Plasmodium falciparum* were used in this study: F32 (Tanzania), Dd2 (SE Asia), K1 (SE Asia), FcB1 (Colombia), K14 (Cambodia) and 3D7 (Africa). In the routine culture conditions of laboratory A (Marseille), that is in an incubator with a fixed atmosphere of 5% CO_2_, 10% O_2_ and 85% N_2_ at 37°C, strain 3D7 was CQ-sensitive (IC_50_ < 100 nM), whereas Dd2, K1 and K14 were CQ-resistant (IC_50_ > 100 nM). In the routine culture conditions of laboratory B (Paris), that is in a candle jar with an atmosphere of approximately 17% O_2_, 3% CO_2_ and 80% N_2_ at 37°C, 3D7, F32 and Dd2 were CQ-sensitive, whereas K1, K14 and FcB1 were CQ-resistant. Cultures were grown in complete medium consisting of RPMI 1640 (Life Technologies Inc.) supplemented with 11 mM glucose, 27.5 mM NaHCO_3_, 100 UI/mL penicillin, 100 *μ*g/mL streptomycin, and 8–10% heat-inactivated human serum, following the procedure of Trager and Jensen [32]. Parasites at the ring stage (laboratory A) or asynchronous parasites (laboratory B) were grown in human A+ or O+ red blood cells at a 1.5% (laboratory A) or 2% (laboratory B) haematocrit and a 0.8–1% parasitaemia. Synchronization of the parasites was performed by sorbitol treatment [33].

Stock solutions (1 mM) of compounds **1**–**5** were prepared in DMSO and stored frozen at -20°C. Further dilutions were in complete culture medium. The complexes were tested for their inhibitory effect toward *P*. *falciparum* intraerythrocytic development. Decreasing concentrations of the compounds and CQ or AQ were established by twofold serial dilutions (maximum DMSO concentration was 0.5% v/v) and distributed (100 *μ*L/well) in a 96 well microplate; DMSO was distributed as a control. 100 *μ*L from a culture at a 3% (laboratory A) or 4% (laboratory B) hematocrit in complete medium was added per well. At time zero (laboratory A) or after 24 h of the incubation (laboratory B), 1.0 *μ*Ci (laboratory A) or 0.5 *μ*Ci (laboratory B) of ^3^H-hypoxanthine was added per well. Then the culture proceeded until the parasite cycle was completed (i.e. to 42 h or 48 h according to the strain). Plates were freeze-thawed and harvested on filters. Dried filters were moistened in scintillation liquid mixture (OptiScint, Hisafe) and counted in a 1450 Microbeta counter (Wallac, PerkinElmer). The percentage of growth inhibition was calculated from the parasite-associated radioactivity. 100% ^3^H-hypoxanthine incorporation was determined from a control grown in the absence of drug or test compound. Antimalarial activity was determined as the concentration of drug inducing 50% of growth inhibition (IC_50_) according to Desjardin et al. [34] or by nonlinear regression analysis from the dose-response relationship as fitted by Riasmart software (Packard).

#### Cytotoxicity measurements

The rat L-6 and human MRC-5 cell lines were routinely grown in RPMI 1640 supplemented with 11 mM glucose, 27.5 mM NaHCO_3_, 100 UI/mL penicillin, 100 *μ*g/mL streptomycin and 10% fetal calf serum (RPMI-FCS), in a 5% CO_2_ incubator at 37°C. For cytotoxicity assays, 100 μL per well of a 2×10^4^ cells/mL suspension in RPMI-FCS were deposited in a 96-well microplate and incubated overnight in culture conditions. Then decreasing concentrations of the compounds to test were established by twofold serial dilutions in RPMI-FCS and distributed at a rate of 100 *μ*L per well and the microplate was put back in the incubator for an additional three (L-6) or five (MRC-5) day period. Then the supernatant in each well was discarded and replaced by 100 *μ*L of MTT 1 mg/mL in RPMI-FCS. The microplate was re-incubated in culture conditions for three hours, then 100 *μ*L of 10% SDS was added by well. The cells were left overnight in the incubator then the OD at 540nm was measured with the Bio-Tek FL600^™^ microplate reader equipped with the KC4^™^ software. Cytotoxic activity was expressed as the concentration of drug inducing 50% of growth arrest (CC_50_), with 100% growth being determined from cells grown in the absence of test compounds. The selectivity of each compound to *P*. *falciparum* was assessed through the selectivity index (SI), defined as CC_50_ (to L6 or MRC5 cells) / IC_50_ (to *P*. *falciparum*).

#### *In vitro* microsomal metabolic stability measurements

The evaluation of microsomal stability was performed by Cyprotex US, LLC, Watertown, MA. Samples were analyzed by LC/MS/MS using an Agilent 6410 mass spectrometer coupled with an Agilent 1200 HPLC and a CTC PAL chilled autosampler, all controlled by MassHunter software (Agilent). After separation on an HILIC HPLC colum (Sepax HILIC 3 *μ*M 2.1 x 30 mm) using an acetonitrile-water gradient system, peaks were analyzed by mass spectrometry (MS) using ESI ionization in MRM mode. The signal was optimized for each compound by ESI positive or negative ionization mode. An MS2 scan or an SIM scan was used to optimize the fragmenter voltage and a product ion analysis was used to identify the best fragment for analysis, and the collision energy was optimized using a product ion or MRM scan. An ionization ranking was assigned indicating the compound’s ease of ionization.

Each test compound (1 *μ*M) was incubated in duplicate with human liver microsomes at 37°C. The reaction contains microsomal protein (0.3 mg/mL) in 100 nM potassium phosphate, 2 mM NADPH, 3 mM MgCl_2_, pH 7.4. A control reaction omitting NADPH was performed for each compound in order to detect any NADPH-independent degradation. Aliquots were removed at 0, 10, 20, 40, and 60 min and mixed with an equal volume of ice-cold Stop Solution (methanol containing haloperidol, diclofenac, or other internal standard). The stopped reactions were further incubated for at least 10 min at -20°C, and an additional volume of water was added. The samples were centrifuged to remove precipitated protein, and the supernatants were analyzed by LC/MS/MS to quantify the remaining parent. Data were converted to % remaining by dividing by the time zero concentration value, and fitted to a first-order decay model to determine half-life values. Intrinsic clearance values were calculated from the half-life and protein concentrations by using the equation CL_int_ = ln 2 / t_1/2_ [microsomal protein].

### Computational details

All electronic structure calculations were performed at the B3LYP level of theory in combination with 6-31G(d) basis set as implemented in the Gaussian09 suite of quantum chemical programs [35–38]. An ultrafine grid was used for all calculations. The effect of solvation was modeled using a polarizable continuum model and atomic radii form Truhlar and co-workers in both water and 1-Octanol continuum [39]. All structures were confirmed to be minima via frequency calculations in both gas- and solvent-phases. We optimized the neutral, monoprotonated, and diprotonated forms of all molecules. Molecular orbital surfaces are generated for gas phase optimized geometry using an iso-surface value of 0.02. All molecular docking simulations were performed with Autodock4.2.11 [40], with default force field, which includes parameters for heme Fe, as well as for C/H/N/O/Cl. The structure of monomeric heme used was from CYP51 (PDB ID: 4H6O; http://www.rcsb.org/pdb/explore/explore.do?structureId=4H6O), and heme dimers from Pagola et al. [41]. For the ligands, the B3LYP/6-31G(d) optimized geometries of the diprotonated molecules (AQ, CQ, **1**–**5**) were used as starting points for all docking simulations. All acyclic bonds in the ligands were rotatable for docking calculations. A grid box with a spacing of 0.375Å, size of 60 x 60 x 60, and centered at the rigid heme receptor was used to generate atomistic grid by AutoGrid4.2. Molecular docking was performed using Lamarckian genetic algorithm (LGA) with search parameters set for 156 GA runs, with a population size of 200, a maximum number of 2.5 × 10^5^ energy evaluations, a maximum number of 2.7 × 10^4^ generations, a mutation rate of 0.02, and a crossover rate of 0.8. Other docking parameters were set to default. The docked conformations were clustered into groups of similar binding modes using a root mean square deviation clustering tolerance of 2.0Å.

## Supporting Information

S1 Fig Docked poses of all molecules studied on a heme receptor. The aminoquinoline molecules were employed in the diprotonated form. Only polar hydrogens were shown for improved clarity. Atom color code: white: H, brown: C, blue: N, red: O, and gold: Fe.
(TIF)

S2 Fig HOMO and LUMO plots of all the systems. Computed at the B3LYP/6-31G(d) level at isosurface value of 0.02.
(TIF)

S1 Table Additional Antiplasmodial Activity Data from Lab. B. (DOCX)

S2 Table B3LYP/6-31G* optimized geometry and energy (in Hartrees) of all molecules studied. (DOCX)
