# Supplementary figures and images for: Synthesis of New 4-Aminoquinolines and Evaluation of Their In Vitro Activity against Chloroquine-Sensitive and Chloroquine-Resistant Plasmodium falciparum
Source: PLoS One. 2015 Oct 16;10(10):e0140878. doi: 10.1371/journal.pone.0140878 (PMC4608832; doi:10.1371/journal.pone.0140878)

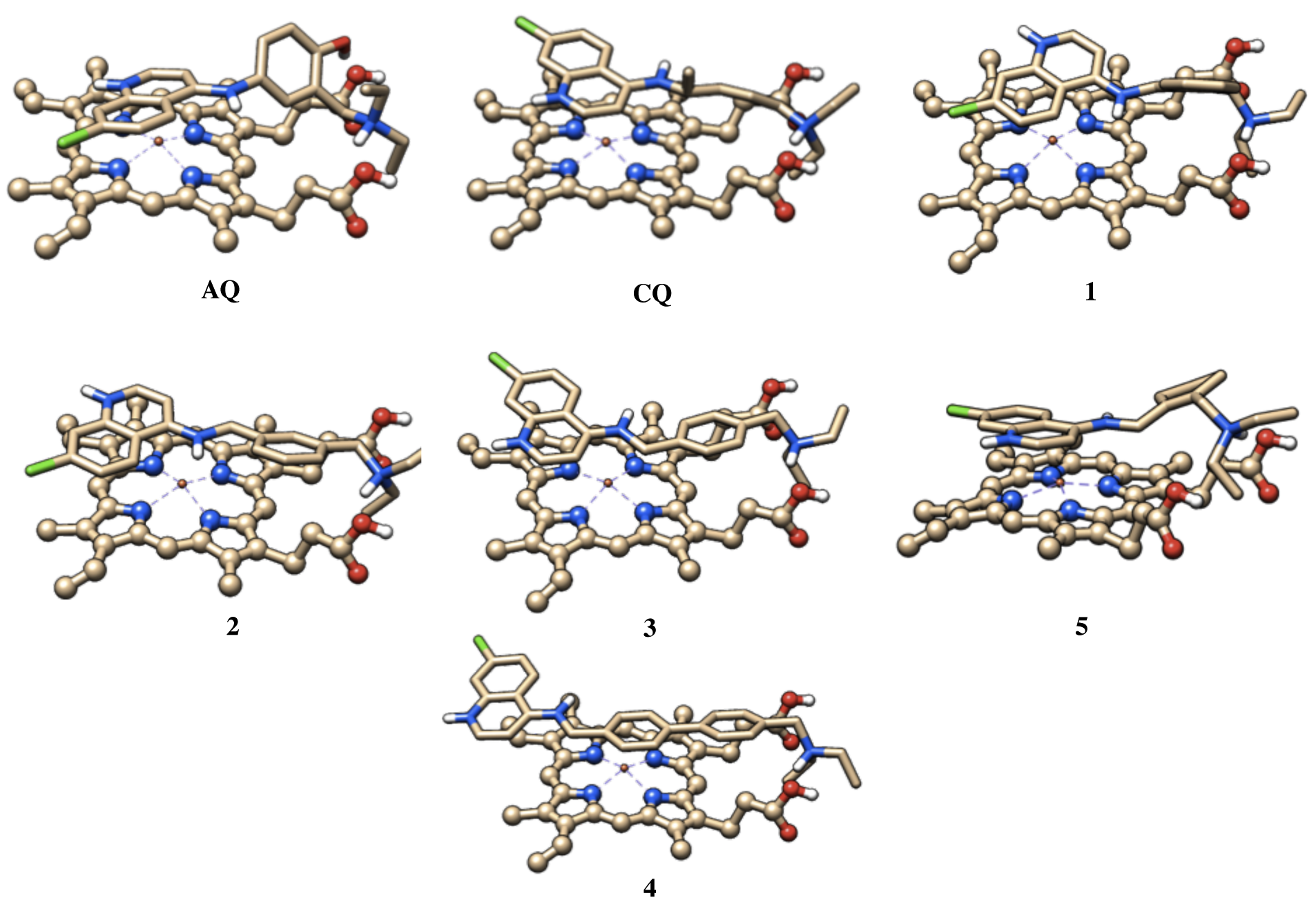

Supplement: S1 Fig — The aminoquinoline molecules were employed in the diprotonated form. Only polar hydrogens were shown for improved clarity. Atom color code: white: H, brown: C, blue: N, red: O, and gold: Fe. (TIF) [file pone.0140878.s001.tif]

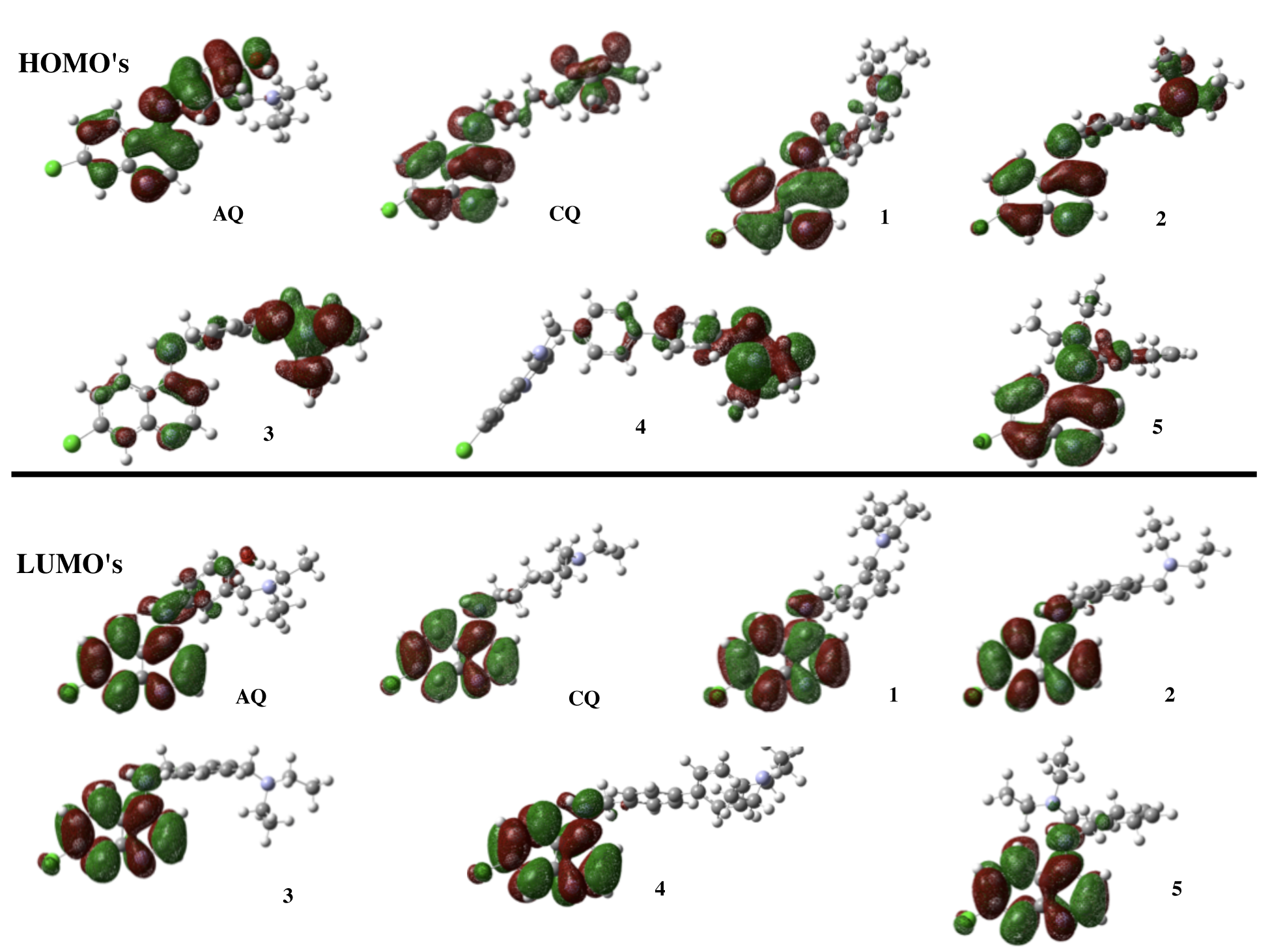

Supplement: S2 Fig — Computed at the B3LYP/6-31G(d) level at isosurface value of 0.02. (TIF) [file pone.0140878.s002.tif]
