# Supplementary material for: Synthesis of New 4-Aminoquinolines and Evaluation of Their In Vitro Activity against Chloroquine-Sensitive and Chloroquine-Resistant Plasmodium falciparum
Source: PLoS One. 2015 Oct 16;10(10):e0140878. doi: 10.1371/journal.pone.0140878 (PMC4608832; doi:10.1371/journal.pone.0140878)
Supplement: S1 Table — (DOCX) [file pone.0140878.s003.docx]

**S1 Table. Additional antiplasmodial activity data.**

|  | Antiplasmodial activity, IC_50_ (nM) | | | |
| --- | --- | --- | --- | --- |
|  | F32 | K14 | FcB1 | |
| CQ | 33.9±12.32 | 103.0±92.53 | | 111±43 |
| **1** | 30.0±15.18 | 13.5±4.66 | | 12.1±1.2 |
| **2** | 14.7±0.45 | 23.6±1.73 | | 30.3±1.7 |
| **3** | 24.3±2.90 | 33.0±5.76 | | 31.5±5.7 |
| **4** | 16.9±1.00 | **7.50±1.08** | | 17.9±1.7 |
| **5** | 25.4±5.31 | 18.7±1.16 | | - |

IC_50_ ± SD values (nM) determined from independent experiments performed in triplicate under the conditions of laboratory **B** (Paris). CQ: chloroquine, -: not determined.
