# Supplementary material for: Synthesis of New 4-Aminoquinolines and Evaluation of Their In Vitro Activity against Chloroquine-Sensitive and Chloroquine-Resistant Plasmodium falciparum
Source: PLoS One. 2015 Oct 16;10(10):e0140878. doi: 10.1371/journal.pone.0140878 (PMC4608832; doi:10.1371/journal.pone.0140878)
Supplement: S2 Table — (DOCX) [file pone.0140878.s004.docx]

**S2 Table. B3LYP/6-31G* optimized geometry and energy (in Hartrees)**

**of all molecules studied**

| **Amodiaquine** (E= -1475.060681)  N 4.94961100 -0.60062600 0.60386500  C 3.62548600 -0.28555300 1.19410900  C 4.89800800 -1.75759200 -0.31685800  C 5.97007800 -0.80624800 1.65653900  C 2.72784900 0.58472000 0.32982900  H 3.81934000 0.25731700 2.12800900  H 3.09047800 -1.20751500 1.47906100  C 4.14906700 -1.51063000 -1.62671700  H 5.93020300 -2.02364300 -0.55920300  H 4.46176100 -2.62737600 0.20865300  C 7.40851300 -0.69334200 1.15023800  H 5.81301600 -0.03142200 2.41392600  H 5.81938500 -1.77780300 2.16101500  C 3.26259700 1.62991700 -0.45540600  C 1.34157500 0.43665800 0.40712000  H 4.27560000 -2.37995600 -2.28183900  H 3.07771300 -1.35942100 -1.47418300  H 4.54638200 -0.63121700 -2.14335700  H 8.09979600 -0.75964500 1.99767900  H 7.56960500 0.26914100 0.65272400  H 7.67834700 -1.48928600 0.44924100  C 2.39615300 2.49748300 -1.13084600  O 4.59985700 1.82205300 -0.58347800  C 0.47283700 1.29118300 -0.28304300  H 0.92552000 -0.34678000 1.03534200  C 1.01811600 2.32835300 -1.04999600  H 2.82737900 3.29313500 -1.73023000  H 5.03456700 1.00793800 -0.20804000  N -0.93528100 1.17173800 -0.15660500  H 0.35852400 2.99751800 -1.59767700  C -1.69428500 0.03276100 -0.34644000  H -1.43980300 2.04468100 -0.20909700  C -3.10492500 0.06996700 -0.05488200  C -1.17168800 -1.16776700 -0.81355300  C -3.76789100 1.19901700 0.48929100  C -3.87016600 -1.11007900 -0.32243800  C -2.02955100 -2.26552800 -1.01200900  H -0.12075500 -1.25538600 -1.06117400  C -5.12542600 1.19307500 0.72623200  H -3.20407500 2.08509200 0.76704200  C -5.26963700 -1.09577300 -0.07915400  N -3.33214000 -2.27145700 -0.79653200  H -1.60453800 -3.19468400 -1.39240100  C -5.87054700 0.03266300 0.42410200  H -5.62176900 2.05879700 1.15063300  H -5.83148600 -1.99636800 -0.29770100  Cl -7.60449100 0.04548000 0.72397600 | **Chloroquine** (E= -1365.3408245)  C -2.48299100 -0.78490700 2.51667500  C -4.06496400 0.05260900 1.11050800  C -3.30158200 -0.28767500 -0.05160600  C -2.00086900 -0.89160900 0.14340900  C -1.62635600 -1.14737000 1.46067200  H -5.88464300 0.93301500 1.84634300  H -2.15908400 -0.98912400 3.53762200  C -5.33065400 0.67740400 0.95065100  C -3.86526800 -0.02415300 -1.32595100  H -0.67451300 -1.60147900 1.69860200  C -5.09855400 0.57561300 -1.46426800  C -5.82151800 0.93298300 -0.30658800  H -3.34790300 -0.32359400 -2.23321600  H -5.51981800 0.76483600 -2.44542400  Cl -7.39055400 1.70647700 -0.49879900  C 0.13679500 -1.72932200 -1.00312600  C 5.09765000 -0.49448100 -0.50570500  H 5.29670100 -1.45841200 -0.02088500  H 5.21607300 -0.66452200 -1.59649500  C 7.41407200 -0.09862100 0.14225200  H 7.90692200 -0.23098200 -0.84269300  H 7.30650400 -1.10175200 0.57147300  C 6.05250100 1.75582500 -0.71213800  H 7.04853900 1.95878300 -1.13187700  H 5.37944400 1.68348400 -1.57876300  C 5.62217000 2.93362200 0.16691900  H 4.61451800 2.77574000 0.56553700  H 6.29939600 3.05291400 1.01886200  H 5.62285600 3.86893900 -0.40722500  C 8.32329900 0.72374200 1.05704000  H 9.29511500 0.22878900 1.16788300  H 8.50965300 1.72692300 0.65817000  H 7.87076700 0.83161000 2.04845000  N 6.07031500 0.46937200 0.00865300  N -1.23555900 -1.19496000 -0.95677900  H -1.50461800 -0.71012500 -1.79865900  N -3.65559100 -0.19448200 2.38943300  H 0.34835900 -1.78882100 -2.07899100  C 1.18349200 -0.76375200 -0.40090600  C 2.63896500 -1.15345200 -0.69731300  C 3.64493600 -0.10060700 -0.21160900  H 2.87311300 -2.11724100 -0.22521400  H 2.76363800 -1.30487500 -1.78052100  H 3.41213000 0.86296700 -0.68468700  H 3.53982900 0.05160300 0.87050500  H 0.98919200 0.23615600 -0.81253300  H 1.04248700 -0.67828200 0.68292500  C 0.21633200 -3.16884500 -0.46978200  H 1.17937600 -3.61612200 -0.73476700  H 0.11273300 -3.22490300 0.61732500  H -0.57623500 -3.77715900 -0.91610800 |
| --- | --- |
| **Compound 1** (E= -1439.1396823)  C -2.44297100 -0.17295400 2.66992400  C -4.02712800 -0.19356500 1.03069700  C -3.05177600 -0.39306000 0.00212100  C -1.66553300 -0.45163100 0.39671100  C -1.38637700 -0.34306100 1.75550100  H -6.11965000 0.05274300 1.46832200  H -2.20317700 -0.08617000 3.72981700  C -5.39932600 -0.10526300 0.67417900  C -3.49834200 -0.53001500 -1.33651700  H -0.36658400 -0.34798100 2.12159800  C -4.83425900 -0.44699700 -1.66438600  C -5.78005600 -0.22457300 -0.64045500  H -2.79217200 -0.73294400 -2.13703500  H -5.16497500 -0.55824200 -2.69110300  Cl -7.48108400 -0.11415600 -1.07686700  C 0.73098000 -0.74758100 -0.26574000  H 0.84293800 -1.40405800 0.60509200  C 3.64210900 -0.76095600 0.43345700  H 3.62937800 -1.02474300 1.50031000  H 3.12463200 -1.59005800 -0.08302600  C 5.82872900 -1.76783700 0.60574200  H 5.51108600 -2.75123800 0.20151500  H 5.60505100 -1.78658700 1.67918800  C 5.17886600 -0.58133100 -1.44988400  H 5.46083600 -1.56282000 -1.86958300  H 4.19435700 -0.34389400 -1.86851600  C 6.16166900 0.49565800 -1.91803700  H 5.83172200 1.48312900 -1.58075800  H 7.17111700 0.32856400 -1.53277000  H 6.21490000 0.50783700 -3.01380900  C 7.34036000 -1.62424200 0.43124900  H 7.85179700 -2.41658000 0.98962000  H 7.64722000 -1.71258300 -0.61573000  H 7.68426900 -0.65633800 0.81094500  N 5.04562700 -0.67412800 0.01913500  N -0.68719600 -0.63635300 -0.55111100  H -0.93256700 -0.43339600 -1.50752500  N -3.72212300 -0.08783700 2.35723900  H 1.17701900 -1.29693900 -1.10446100  C 1.49434000 0.56201900 -0.04792200  C 2.87314200 0.54332000 0.25446800  C 0.83656600 1.79194800 -0.15569300  C 3.54057900 1.75796100 0.45363600  C 1.51539700 2.99506000 0.03689500  H -0.22351200 1.80759300 -0.38756900  C 2.87403000 2.97774600 0.34605800  H 0.98087500 3.93716800 -0.05036600  H 3.41417600 3.90735200 0.50511400  H 4.59954600 1.72517200 0.68860400 | **Compound 2** (E= -1439.1443163)  C 2.36185400 2.43557400 -0.12260200  C 4.20869900 1.10794500 0.03785900  C 3.47138000 -0.06763400 -0.31218300  C 2.07239900 0.08842600 -0.62088000  C 1.52982600 1.36210600 -0.49724700  H 6.13665100 1.90016200 0.57369200  H 1.92120000 3.42906400 -0.03769700  C 5.59663300 0.99599500 0.31797800  C 4.15006700 -1.31244700 -0.32998700  H 0.47736900 1.54390100 -0.67708600  C 5.49595200 -1.40433300 -0.04832700  C 6.21359700 -0.23063600 0.26794800  H 3.60987900 -2.23253300 -0.53580200  H 6.00408100 -2.36228300 -0.05815300  Cl 7.93305800 -0.36496400 0.61675400  C -0.02480500 -0.88545400 -1.54291400  H -0.07353300 -0.08003200 -2.29193800  C -4.53734500 1.04089500 -0.31271300  H -4.47590800 2.00514900 0.20943500  H -4.49842900 1.27665700 -1.39485000  C -6.90229700 1.38698800 0.06130300  H -7.13967400 1.71052200 -0.97312600  H -6.55548700 2.28102800 0.59362300  C -6.08996500 -0.80968100 -0.69290800  H -6.88806900 -0.61749600 -1.42969500  H -5.19888600 -1.07135100 -1.27480900  C -6.45892000 -2.00943300 0.18414600  H -5.63411300 -2.24994400 0.86235300  H -7.34670000 -1.81243700 0.79214600  H -6.66147400 -2.88990500 -0.43860300  C -8.18151600 0.88912500 0.73405000  H -8.92169500 1.69683400 0.76642400  H -8.63419000 0.05205300 0.19267900  H -7.97736400 0.56439800 1.75984800  N -5.80324000 0.41682400 0.07886600  N 1.32357700 -1.00654800 -1.00145100  H 1.85522300 -1.77914800 -1.37247500  N 3.65423100 2.35260200 0.12797100  H -0.22379500 -1.82028800 -2.08119900  C -1.11226600 -0.67067300 -0.49924000  C -2.26751000 0.04212900 -0.84428400  C -1.01293000 -1.21888800 0.78179900  C -3.31949300 0.21368100 0.06029000  H -2.34978800 0.47521900 -1.84079400  C -2.05681100 -1.05498700 1.69481900  H -0.11601800 -1.76350400 1.06161500  C -3.20324200 -0.34659200 1.33938100  H -1.97185000 -1.48102000 2.69130800  H -4.02121900 -0.22342400 2.04285900 |
| **Compound 3** (E= -1439.1444619)  C 1.86889700 -1.75073900 1.47847500  C 3.79766000 -1.05979200 0.47760800  C 3.29343200 0.26454600 0.27761600  C 1.97351900 0.55923300 0.77551300  C 1.26471600 -0.48341800 1.36070200  H 5.47106400 -2.38345000 0.19571500  H 1.30144200 -2.55494100 1.94713600  C 5.10688000 -1.37672300 0.02718200  C 4.11022100 1.20509800 -0.39967500  H 0.25377500 -0.34172500 1.72261200  C 5.37576000 0.87874600 -0.83684100  C 5.86770000 -0.42414500 -0.60648500  H 3.73794600 2.20210600 -0.61896400  H 5.98982900 1.60361200 -1.35977100  Cl 7.48876500 -0.82137500 -1.16394600  C 0.23155500 2.25396300 1.31515700  H 0.23040400 1.92464600 2.36574400  C -4.82028500 0.69918700 -1.22825800  H -5.47017100 1.58427400 -1.19948700  H -4.64724700 0.48238700 -2.30095000  C -6.94067100 -0.43208700 -0.95671100  H -7.04205100 -0.74447800 -2.01648900  H -7.33255200 0.59063700 -0.89646200  C -4.84036000 -1.68208400 -0.67344600  H -5.37283600 -2.32883100 -1.39101300  H -3.84833600 -1.50626300 -1.10429900  C -4.66021700 -2.41088900 0.66111000  H -4.06394200 -1.80133000 1.34754200  H -5.61866300 -2.62058400 1.14491200  H -4.14047000 -3.36486900 0.50750500  C -7.81223900 -1.33630000 -0.08512500  H -8.86102900 -1.24625700 -0.39058100  H -7.53423100 -2.39122300 -0.17690700  H -7.73224900 -1.05064800 0.96913300  N -5.53399600 -0.38350500 -0.54624500  N 1.45420000 1.83092900 0.64228400  H 2.13950300 2.56124800 0.52272600  N 3.08740700 -2.05939200 1.07849000  H 0.25720200 3.35047000 1.33546400  C -1.05474900 1.79735400 0.64217900  C -2.20270300 1.57378700 1.41376900  C -1.14060100 1.63792500 -0.74310900  C -3.40744700 1.21068500 0.81588800  H -2.15293500 1.68441600 2.49570300  C -2.34631300 1.26527700 -1.33958500  H -0.25416900 1.78812500 -1.35254800  C -3.49681200 1.05174800 -0.57346400  H -2.39119100 1.13568800 -2.41895600  H -4.28905100 1.02593100 1.42262500 | **Compound 4** (E= -1670.2018951)  C -4.15015500 -0.48150600 2.41838200  C -5.69592400 0.55656000 1.10214800  C -5.18365600 -0.05164800 -0.08770200  C -4.08590000 -0.97446500 0.05328000  C -3.56688600 -1.15895800 1.32925200  H -7.17423200 1.89045500 1.91889300  H -3.73513100 -0.64355900 3.41328700  C -6.79799800 1.44714700 1.00432100  C -5.77477700 0.28869500 -1.33079900  H -2.71795200 -1.80904900 1.50183300  C -6.83903600 1.16074800 -1.40861400  C -7.34957500 1.72977400 -0.22194100  H -5.37494900 -0.10964100 -2.25933300  H -7.27958400 1.41968800 -2.36508000  Cl -8.71403400 2.83458200 -0.33878900  C -2.63394500 -2.72407700 -0.96017400  H -2.94744100 -3.43873600 -0.18347000  C 7.15682300 -0.24929800 0.45567000  H 7.44478500 -0.45187900 1.50636500  H 7.74533400 -0.94437500 -0.15852800  C 6.91566100 2.14285600 0.90165000  H 7.69070600 2.58291300 1.55166800  H 6.19597900 1.66141300 1.57323700  C 8.95821100 1.25103100 -0.14059600  H 9.48836200 1.19023400 0.83224500  H 9.30182100 0.39524800 -0.73448300  C 9.37591600 2.53559200 -0.85640400  H 8.86712400 2.62239800 -1.82233800  H 9.14811000 3.42967800 -0.26705700  H 10.45762100 2.52663500 -1.03319700  C 6.18586800 3.24539000 0.12930100  H 6.85253700 3.77040500 -0.56107400  H 5.36550700 2.81773000 -0.45572500  H 5.76536000 3.98343400 0.82384600  N 7.50934400 1.10779700 0.03033000  N -3.57911100 -1.61811800 -1.05787100  H -4.21205600 -1.67840100 -1.84087900  N -5.18140600 0.33779700 2.34801200  H -2.69127200 -3.26113600 -1.91506500  C 1.53344800 -1.62369800 -0.31954100  C 0.65885000 -0.76855200 -1.01166500  C -0.67854100 -1.10481500 -1.20599200  C -1.19248900 -2.30701500 -0.70933500  C -0.33051500 -3.16080100 -0.01209000  C 1.00935800 -2.82908300 0.17586600  C 2.95887700 -1.26385700 -0.11469700  C 3.68997700 -0.59484800 -1.11298800  C 5.02605700 -0.25644700 -0.92258400  C 5.68538400 -0.57231500 0.27332900  C 4.96129500 -1.23331700 1.27041600  C 3.62269200 -1.57594000 1.08170400  H 1.02593000 0.18561200 -1.37918900  H -1.33975400 -0.42031000 -1.72936100  H -0.70770800 -4.10266700 0.38227000  H 1.66257400 -3.52204700 0.69865800  H 3.21023900 -0.36151600 -2.05977600  H 5.57597700 0.26239900 -1.70235900  H 5.44714100 -1.47606600 2.21322600  H 3.07765000 -2.06200300 1.88629000 |
| **Compound 5** (E= -1440.3139899)  C 4.44797000 0.20396700 0.22381300  C 4.10181500 -1.08951500 0.53249700  C 2.82009200 -1.58899400 0.17692200  C 1.89358500 -0.71924300 -0.48055000  C 2.30987500 0.59631800 -0.80493000  C 3.56147200 1.06214300 -0.46156900  H 4.78483400 -1.76067000 1.04008400  C 0.58425100 -1.23901900 -0.79679000  H 1.65080800 1.24939000 -1.36847900  H 3.87519000 2.06684000 -0.72292100  C 0.35144800 -2.57829700 -0.49126800  C 1.35997300 -3.33785900 0.13020300  H -0.60724500 -3.04180800 -0.69164900  H 1.15126600 -4.38117200 0.36902300  N -0.34202400 -0.42528600 -1.40185100  H -0.31014500 0.56688200 -1.17728300  Cl 6.03840600 0.81732900 0.66660900  N 2.55234200 -2.89286300 0.48176900  C -1.69995700 -0.85229700 -1.68473000  H -2.11246900 -0.12680900 -2.39901400  H -1.65009700 -1.80219500 -2.23059200  C -2.68081500 -1.01304900 -0.52291100  C -2.72232900 -0.25887100 0.59067000  C -3.69542200 -2.10856600 -0.79851400  C -3.75557500 -0.50107000 1.67810000  C -4.74024300 -2.29297400 0.26157700  H -4.18007800 -1.91548400 -1.77209800  H -3.15961800 -3.06133700 -0.95508300  C -4.75960300 -1.57543600 1.38318700  H -4.28303700 0.44522400 1.88718800  H -3.23432600 -0.72875500 2.62449400  H -5.52898900 -1.74909800 2.13359300  H -5.49263600 -3.05966400 0.08475700  C -1.75667400 0.86751300 0.95454000  H -0.77737100 0.43841400 1.20427700  H -2.12003200 1.32949300 1.88950000  N -1.51807200 1.90178100 -0.07322600  C -2.75387200 2.51789500 -0.59371900  C -3.55777900 3.40612200 0.37092300  H -3.39172700 1.70082100 -0.94402100  H -2.48379600 3.10196600 -1.48002800  H -4.46270600 3.76560200 -0.13208000  H -3.87454400 2.86068800 1.26649800  H -2.99119900 4.28531400 0.69639500  C -0.53007100 2.87446500 0.42689600  C -0.08788100 3.90002600 -0.61701800  H 0.34620200 2.29860200 0.74838000  H -0.89675700 3.40058800 1.32708300  H 0.74888900 4.48715500 -0.22361000  H 0.24487600 3.40783200 -1.53827900  H -0.88547000 4.60261400 -0.87930200 |  |
